# “So, you must understand that that group changed everything”: perspectives on a telehealth group intervention for individuals with chronic pain

**DOI:** 10.1186/s12891-022-05467-7

**Published:** 2022-06-04

**Authors:** Dawn Ernstzen, Janet Keet, Kerry-Ann Louw, Jocelyn Park-Ross, Lorien Pask, Cameron Reardon, Maia Zway, Romy Parker

**Affiliations:** 1 Division of Physiotherapy, Department of Health and Rehabilitation Sciences, Stellenbosch University, Cape Town, South Africa; 2 Pain Management Unit, Department of Anaesthesia and Perioperative Medicine, University of Cape Town, Cape Town, South Africa; 3 Department of Psychiatry, Stellenbosch University, Stellenbosch, South Africa; 4 Division of Physiotherapy, Ukwanda Centre for Rural Health Stellenbosch University, Stellenbosch, South Africa; 5 Department of Anaesthesiology and Critical Care, Tygerberg Hospital, Stellenbosch University, Stellenbosch, South Africa

**Keywords:** Telehealth, Chronic pain, Patient experience, Chronic pain management programmes, Qualitative

## Abstract

**Background:**

The Patient Education Empowerment Programme (PEEP) is an interdisciplinary group intervention for people living with chronic pain. As a result of the COVID-19 pandemic, lockdown and restrictions on in-person group-based health care delivery in South Africa, PEEP was modified to a telehealth electronic format (ePEEP) and offered to patients on a waiting list at two interdisciplinary chronic pain clinics in Cape Town, South Africa. The purpose of this study was to explore the feasibility and acceptability of ePEEP through the perspectives of individuals with chronic pain who participated in ePEEP.

**Methods:**

A qualitative, exploratory descriptive study was conducted. One month after completion of the 6-week ePEEP programme, individuals who participated, were recruited for the study. Data were collected through semi-structured interviews. Data analysis followed an iterative process of inductive content analysis.

**Results:**

Six individuals, all women, consented and participated in the study. Three main themes emerged from the data. Theme one focussed on how ePEEP initiated a journey of personal development and discovery within the participants. In theme two, participants reflected on the importance and value of building peer and therapeutic relationships as part of ePEEP. In theme 3, participants shared that the online learning environment had features which influenced learning about pain in different ways.

**Conclusion:**

ePEEP was found to be acceptable, feasible and valuable for the participants. ePEEP facilitated self-discovery, empowerment, relationship building and transformation in the participants, through experiential learning. Although barriers and facilitators to learning were present, both enhanced the learning experience. The positive reception of this telehealth initiative indicates potential for enhanced access to chronic pain management services in the South African setting.

**Supplementary Information:**

The online version contains supplementary material available at 10.1186/s12891-022-05467-7.

## Introduction

The COVID-19 pandemic, and subsequent lockdown, changed many aspects of healthcare delivery [1]. In South Africa, some healthcare services were temporarily suspended during lockdown and services for people with chronic pain were interrupted. The impact of COVID-19 on South Africans with chronic pain remains to be seen but based on other countries’ experiences, it is likely to impact access to care, effective pain management, support and may intensify health care disparities [2–4]. People with chronic pain often experience increased levels of disability over time, with a concomitant reduced potential for recovery. Therefore, it is critical to provide appropriate treatment and rehabilitation early in their health care journey. There is thus a dire need to support people with chronic pain in new and innovative ways while adhering to lockdown regulations. Telehealth innovations are one way to offer such care.

Chronic pain is a complex, multi-dimensional experience, which impacts wellbeing, leading to decreased functioning and quality of life [5, 6]. In South Africa, one in five people suffer from chronic pain [7]. To address this concern, the Groote Schuur and Tygerberg Hospitals in Cape Town, South Africa, offer interdisciplinary chronic pain management clinics. Both these clinics provide a group based chronic pain management program (CPMP), which is branded PEEP (Pain Education Empowerment Program) [8]. PEEP is six-week group program patients attend for two hours per week. PEEP integrates treatment strategies for chronic pain using principles of cognitive behavioural therapy (CBT), education, pain neuroscience education, mental wellbeing and physical activity. PEEP focusses on addressing patients’ understanding and beliefs about their pain, facilitating skill acquisition and behaviour change to increase self-efficacy and thus their capacity to live and cope with pain [9, 10]. During the pandemic in-person PEEP sessions were suspended due to the high risk of COVID-19 infection when patients travel and attend hospital-based group sessions. However, given the risk of worsening disability for patients due to limitations in access to health care, it was imperative to provide alternative access to pain management. We therefore modified PEEP to be delivered via telehealth as an electronic version (ePEEP) via a smart mobile device on which WhatsApp could be used, to address this need.

Telehealth interventions for chronic pain can be as effective and acceptable as in-person therapy to improve pain, physical activity, function, and psychological variables [11, 12]. Additionally, a telehealth group intervention offers the benefit of increasing social participation using a virtual connection platform [13]. However, there is a knowledge gap regarding the efficacy of telehealth group interventions [13, 14], interdisciplinary telehealth [15] and the processes through which change occurs. There is thus a need to explore the acceptability and feasibility of a telehealth group intervention in the South African context. In this paper we report on patients’ perspectives regarding participation in a telehealth intervention (ePEEP), and their perspectives on the acceptability and feasibility of ePEEP in the SA context. In a separate paper we will report on the perspectives of the clinician providers who facilitated ePEEP.

## Methods

### Study design

A qualitative, exploratory descriptive study design with a phenomenological approach [16] was used. The phenomenological approach was chosen to gain insight into the participants’ lived experience of, and their perspectives regarding participating in a telehealth group program for chronic pain. The study received approval from the University of Cape Town and Stellenbosch University Health Research Ethics Committees and the Groote Schuur and Tygerberg Hospital Facility Management and Ethics committees.

### Population and sample

Patients with chronic pain on the waiting list for PEEP were eligible to participate and were invited to take part in ePEEP. Purposive sampling was used [17], using the criteria as outlined in Table 1. All patients who had completed the first week of the program were eligible since week 1 of ePEEP is foundational. ePEEP utilises synchronous and asynchronous learning in the flexible program, and thus attendance at scheduled activities was not compulsory for inclusion in the study.

**Table 1:** Inclusion and exclusion criteria for participation

Inclusion Criteria	Exclusion Criteria
Adults diagnosed with chronic non-cancer pain and referred to PEEP.^a^	Pending investigations e.g. magnetic resonance imaging, nerve conduction studies
Evaluated as safe to engage in moderate to vigorous physical activity according to the ACSM’s Guidelines for Exercise Testing and Prescription (American College of Sports Medicine, 2013).^a^	Communication impairments limiting ability to engage through electronic platforms
Access to and ability/confidence to work with a smartphone.^b^	

^a^Patients referred to the Chronic Pain Management Clinic have been assessed by a physician to determine eligibility for safe participation in the group and moderate physical activity. The ACSM Guidelines for Exercise Testing and Prescription (American College of Sports Medicine, 2013) guidelines include the following contra-indications for exercise prescription: recent acute myocardial infarction, unstable angina, ventricular tachycardia and other dangerous dysrhythmias, dissecting aortic aneurysm, acute congestive heart failure, severe aortic stenosis, active or suspected myocarditis or pericarditis, thrombophlebitis, recent systemic or pulmonary embolism, acute infection

^b^This criterion was needed since the aim of the study was to determine the acceptability and feasibility of ePEEP and the challenges with technology were investigated. We acknowledge there may be potential participants who do not have access, or who are unable to operate a smartphone. Those patients may be included in follow up programs and alternative measures will be initiated to address the barriers to access

### Instrumentation

An interview guide was developed based on similar research [13, 18, 19]. The interview schedule comprised questions about expectations and experiences regarding participation in ePEEP, challenges experiences as well as potential solutions, participant roles and suggestions for optimising ePEEP. The interview schedule is available as additional file 1. DE, who was not involved in the practical delivery of ePEEP, conducted the interviews in Afrikaans or English, according to the preference of the participant.

### Procedure

Two rounds of ePEEP were conducted with three groups of participants. In the first two groups, patients were allocated into ePEEP groups according to language preference; and the third group was a mixed language group (Afrikaans and/or English). Each ePEEP group had a dedicated facilitator who co-developed e-PEEP. One team member contacted eligible patients to offer them participation in ePEEP (MZ). After completion of the ePEEP intervention, MZ contacted the participant to ask permission to provide their contact details to another team member (DE), for the purpose of being invited to participate in the research study. DE invited those who agreed by sending a text message, complemented by a voice note. Information on the study, its purpose, and an invitation to participate was sent. The participant could provide written informed consent via text message or email. On agreement to participate, an appointment was scheduled for a telephonic interview. Individual semi-structured telephonic interviews were conducted to assure privacy and encourage in-depth discussion. Study participants’ socio-demographic data were obtained after the interview using a short telephonic survey, to enable a description of context. Interviews lasted 30 – 60 min and were audio recorded.

### The intervention (ePEEP)

The telehealth intervention was 6-weeks in duration. The therapeutic components of ePEEP were derived from PEEP and adapted for delivery using smart mobile devices. The research team identified the key educational and skill activities for inclusion through a collaborative process. Thereafter, these were packaged into discrete learning tasks using various forms of multimedia. Table 2 provides a summary of the ePEEP content and delivery. Educational material in the form of an existing workbook in English or Afrikaans were couriered to participants. Educational content such as short videos, podcasts, voice notes, pictures (infographics) and text messages were delivered via WhatsApp at the beginning of each week for asynchronous learning. An online WhatsApp video call session was held weekly via a group call, to facilitate collaborative (synchronous) discussions. At the end of each week, further educational content was shared to expand on the synchronous discussions. ePEEP was delivered using low data methods focussing on mobile messages, via WhatsApp. This was necessary since 60.1% of South Africans access the internet via their mobile devices. However, only 10.4% of South Africans have access to internet connection at home [20]. Participants in ePEEP received a weekly data allowance for 6-weeks, to allow them to access the material sent to them and to allow participation in online facilitated discussions.

**Table 2:** Overview of ePEEP content and delivery

Week	Focus	Front loading (Pre-class)	Class time	Back loading (Follow up)
1	**Introduction, Pain journey, Pain physiology**	Introduction Group rules Pain neuroscience education	Review group rules Share pain story Discuss pain science	Explain pain video Share with family
2	**Goal setting, Exercise as therapy**	Pain in me video Goal setting: theory Goal setting; role modelling video Exercise podcast	Exercise practical (family member present) Reflection on exercise and physical activity Reflection on Goal setting Goal setting task	Goal setting for exercise
3	**Stress and pain, Goal setting**	Stress and Pain Mindfulness theory	Exercise practical Stress and the body exercise Thoughts, feelings, action exercise Sleep hygiene Mindfulness exercise Goal setting	Black dog depression video Mindfulness task Sleep hygiene task Exercise
4	**Nutrition and Goal setting**	Nutrition worksheet task Values clarification worksheet Preparation for class	Reflection on nutrition Raisin mindfulness meditation Nutrition goal setting	Mindfulness task Exercise task
5	**Medication and taking part in health care**	Medication workbook task Take part exercise Write down questions about medication,	Reflection medication and taking part Role play of an interaction with a prescriber Medication goal setting	Exercise Mindfulness exercise Podcast on exercise, mindfulness and eating as medicine
6	**Summary and continuing as self-manager**	Summarising video Practical example: successfully living with chronic pain video Introduce volunteering	Revision of each week Being a self-manager Links to WOW Graduation Closing	

*Front loading* information and tasks were sent to participants prior to their scheduled weekly class time, usually on a Monday. The participant was required to work through the material and complete the tasks. *Scheduled synchronous class time* group interaction with real-time WhatsApp video interaction took place for one hour on a predetermined day (usually Thursdays). The focus was on sharing information, reflecting on the pre-loaded material and sharing feedback on tasks. *Back loading* Relevant follow up information was posted on the e-platform after the class, usually Fridays

With their permission, participants and facilitators were added to a group on the mobile messaging platform on which the intervention was delivered. WhatsApp was chosen as a low data method of delivering a range of content. At the time of conducting the study, this platform was ethically and legally allowed without impacting on patient rights. In the first introductory contact of ePEEP participants completed a statement that they would maintain the confidentiality of the group. In this statement participants specified that they would show mutual respect, and that confidentiality regarding personal information shared in the group would be maintained. Participants could add any additional points regarding conduct in the groups. ePEEP is a group-based program, therefore the information shared in the group cannot be anonymised.

### Data management and analysis

Interview recordings were downloaded to a computer, a unique serial number allocated, and de-identified by DE. Thereafter, the recordings were transcribed by a professional transcription company. Survey data were extracted from the transcriptions and entered into MS Word for descriptive analysis. Inductive, thematic content analysis of the interview transcripts were done [21]. This method involved an iterative process of immersion in data, identification of initial codes, clustering codes into categories and categories into themes [21]. Data were analysed in the language of the interview and translated to English where required for the purpose of this manuscript. The six transcripts were equally divided between DE, RP and KL, who each independently coded two transcripts. The three researchers developed an initial list of codes from the two transcripts allocated to them, then compared and discussed their initial list of codes and emerging themes. Consensus was reached regarding the codes to formulate an initial codebook and preliminary themes. DE applied the amalgamated codebook to the dataset using ATLAS.ti (version 9, Scientific Software Development, GmbH, Berlin, Germany) and added codes where indicated. The latter step was done to be consistent in the application of the codebook to the transcripts. The research team audited the interview analysis, categories and themes and collaboratively identified associations and relationships between themes. Member checking was done by providing the themes and categories to study participants to verify via text message [22].

## Results

### Study participants

**Fig. 1:**
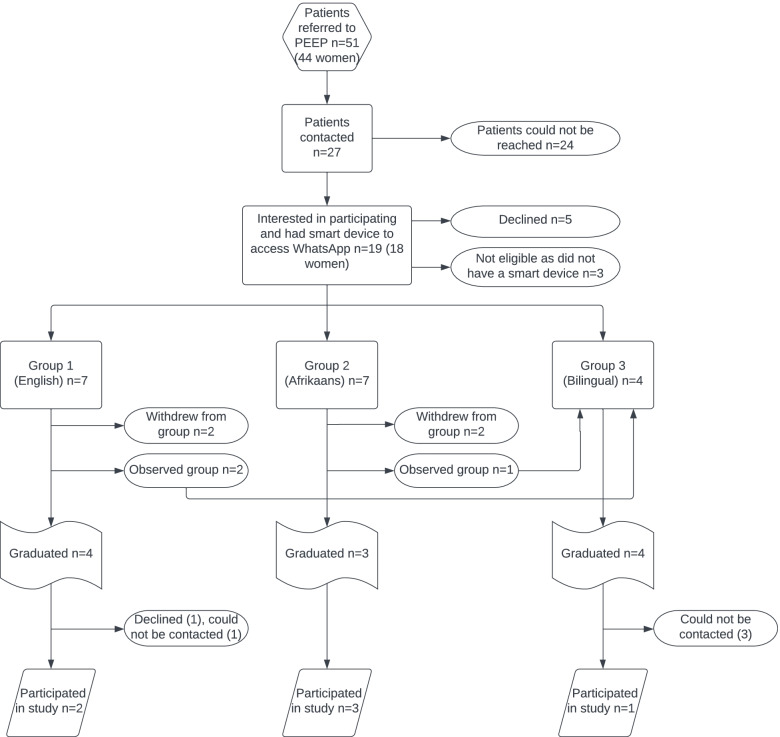
Recruitment and participation in ePEEP

A total of 51 patients were on the waiting lists for PEEP at GSH and TBH and were therefore eligible to be invited to participate in ePEEP. We could only reach 27 out of the 51 via the provided contact details to invite them to take part in ePEEP. Nineteen (18 women, 1 man) indicated that they were interested in participating in ePEEP and had smartphones. Three groups were run with 11 patients (all women) completing the full program to graduation and six agreeing to participate in this study (Fig. 1). One eligible person declined due to her personal circumstances, and four could not be reached telephonically by the facilitators. Table 3 summarizes study participant ages and health conditions. The mean age was 43 years (range: 28 – 52).

**Table 3:** Study participant information

Participant number	Participated in group	Age in years	Health conditions reported
P1	Group 2	48	Low back pain, arthritis, depression, polycystic ovary syndrome
P2	Group 2	52	Fibromyalgia
P3	Group 1	37	Rheumatoid arthritis, Fibromyalgia
P4	Group 1	28	Thoracic Outlet Syndrome
P5	Group 2	44	Post motor vehicle accident, Fibromyalgia, Rheumatoid arthritis
P6	Group 3	51	Post motor vehicle accident, Spastic colon, borderline personality disorder

### Themes

**Fig. 2:**
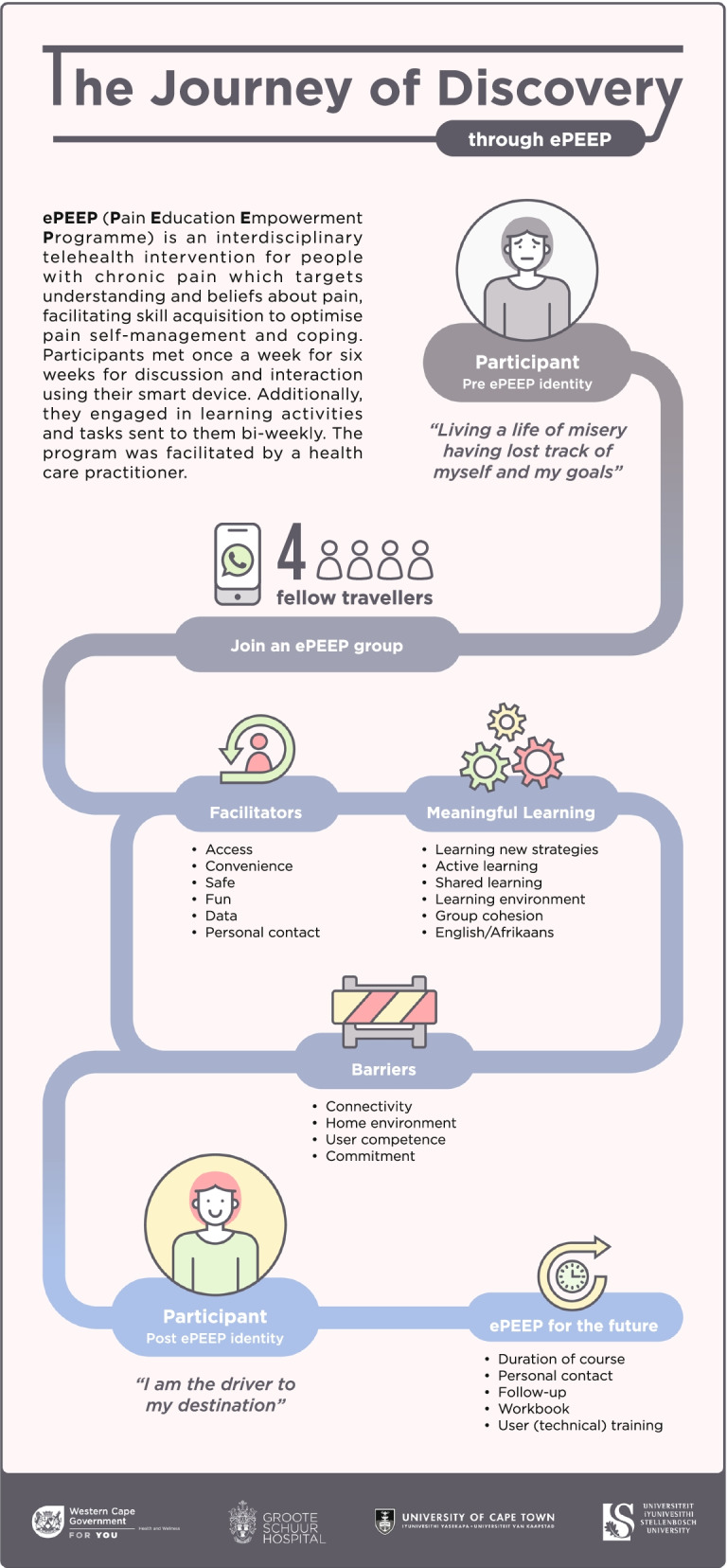
Participants perspectives on ePEEP

Three main themes emerged from the data. In theme one, participants shared their personal journey of discovery and development. Theme two focussed on the affordances of the online learning environment and in theme three, participants reflected on how ePEEP may be optimised. Figure 2 provides a summary of the main themes as well as the associated categories and codes.

#### Theme 1: The journey of discovery

Participants described embarking on a journey of discovery through ePEEP. This journey included elements of self-discovery, empowerment, and transformation via engagement with various role-players on the journey. Participants identified their pre-ePEEP selves, which, through engaging in meaningful learning activities during the journey, shaped their new identity as empowered individuals. Table 4 provides an overview of Theme 1, with substantiating verbatim quotes.

**Table 4:** Theme 1: Journey of discovery during ePEEP

Theme	Subtheme	Category	Code	Substantiating verbatim quote
**THEME 1: The journey of discovery**	Departure point on journey	Identity pre-ePEEP	Identity Role of self Role of family Role of the HCP	*I mean in terms of doing things physically. I can work any power tool a man can work, I can mix cement, everything. So, I thought I did this damage to myself. Gr1_P3* *All you know is you are in a hell of a lot of pain, you cannot get out of bed, you cannot see to your kids, you can’t pick them up, hug them, hold them, play with them because the slightest touch hurts you. You live a life of misery. Gr1_P3* *I experienced the pain, and nobody knew what it was, and they did all this testing I was diagnosed, I had the surgery, and during this time I just lost track of myself, and I lost track of my goals. Gr1_P4* *And I know prior to being in ePEEP I didn’t want this pain. I wanted to stop being this evil that will just snap at people because I was riddled with pain and I was frustrated and moody. And I didn’t want that. I wanted my children to have a happier life, like they had before – I wanted to get back to the person I was before and even better. Gr3_P6*
	Meaningful learning	The learning environment	Open, relaxed Non-judgmental	*Every week he would – before we started the program – he gave us a platform to basically speak about how we feel, how we are dealing with our pain, how we are dealing with our thoughts, so he gave us the, ja – he gave us a platform for us to be heard, Gr1_P4* *And they were of great help to me; that I could share with them what I am going through; and that I did not have to worry about being judged or criticised. Gr2_P1*
		Active engagement/experiential learning Learning new strategies for pain	Exercise Mindfulness Relaxation Nutrition	*There was one of the modules in our program that we did was the physical activity. I don’t think that I would have realised what major impact it has on your body and your life if you start to become physical, it also reduces the amount of pain that you feel and there was a time that I was going through some phase of depression as well, so it actually helped me with that as well. Gr1_P4* *The homework tasks with the exercising, making sure you were fit and posting and reading your book and understanding and meditating – that I loved, especially the meditating part. The relaxation part – I liked that part because nobody would disturb me. Gr1_P3* *I think the other part I found valuable as well was the eating plans, and … because we don’t realise what an important role food plays in our lives. So if you don’t eat properly you won’t be able to function properly, so yes, definitely the eating plans would be number 2 for me. Gr1_P4* *I didn’t just complete my 6-week pain programme, I successfully completed my 6-week pain program. Because I did not stop there and I have got results, and I have learnt that pain is not everywhere, I just need to calm myself down and focus. Gr1_P3* *I also jot down the things I need to say to the doctor, the things I need to ask the doctor so that if there is something that I forget it is written down and that is one of the things that we also learned in the group is that write down your things, prepare yourself before you visit. And one thing I loved, these are things I would normally do and them stating it in the group just affirmed that what I was doing was right. Gr3_P6*
	On reaching the journey destination	Transformed identity Changed mindset An internal locus of control	New life beyond mere existence I am the driver to my destination Decision making	*I am positive. I’m not depressed. I am looking forward to life, I have thanked the pain group on Facebook as well as my fitness group because if it wasn’t for (the facilitator), I’m going to be honest with you – if it wasn’t for (the facilitator). I would not have exercised…, and I would not be climbing a mountain tomorrow. I am more than willing to share my story because I am alive. I am alive, I am not just existing anymore. I am a person. Gr1_P3* *The most important thing that changed for me … my whole life changed for me, all at once. So, I can’t answer that just as in a, like I can sweep my house now. No, everything changed. You need to know that every single thing changed – everything changed. My mindset changed, my body changed, my world changed, my way of thinking changed, my way of doing things has changed – everything has changed. Gr2_P5* *I think that my mindset has changed completely. Like before I started the program, I used to use medication a lot. I don’t use medication at all anymore, so I would still experience some pain but it’s more mind over anything else, so I would do something to distract myself from the pain Gr1_P4* *It is a bit tougher now that I am off the program, but I think that is where self- motivation comes in and you have to realise that you are doing it for yourself and your own well-being and you won’t be, you can’t basically be dependent on somebody else to help you throughout your life. You have to make a decision to do it yourself and you have to stick to the goals that you have set for yourself. Gr1_P4*
		Engaged family	Family support and understanding	*It was nice when you hear the others say they got their family members to do the mindfulness exercises or the walks or the breathing, so just like with my family that implemented with theirs and it became not just something you would share for yourself, but it becomes a family goal and it just makes the, when you achieve things so much greater. Gr3_P6* *I think I have a happier family, they eat healthy, and they participate in the activities. Because I said I am in the program, I can’t just leave now. Gr2_P2*

Three categories of people played a role in participants’ pre-ePEEP identities, namely themselves, their families, and health care providers. Participants attributed their pain to their past activities, leading to self-blame or they attributed their persistent pain to previous traumatic experiences. They reported feeling lost in the healthcare seeking process of enquiry about pain and longed to play a more active role in their family life. Healthcare providers played an important role in validating or non-validating pain and participants’ resultant feelings.

The participants’ journey of discovery was facilitated by the learning activities and the learning environment created during ePEEP sessions. Being part of a group significantly enriched learning. Participants experienced the learning environment as open, relaxed and non-judgmental. The journey of discovery was an active journey with participants valuing learning new skills to enable them to live their lives with pain as a companion. The valued skills acquired on the journey of discovery included exercise, mindfulness, relaxation, and healthy eating.

New knowledge gains and the acquisition of skills enabled a change in participants’ mindset and a sense of empowerment. The participants realized that they were in the driver’s seat to their life’s destination; this required them to shift their locus of control with a newfound determination and self-motivation to set goals and to make decisions. The change in their own perspectives resulted in a change in their family dynamics and the way they approached healthcare providers. Family engagement with the ePEEP learning materials improved the family’s understanding of the person living with pain. Additionally, participants had improved communication with family members which enhanced relationships and redefined roles. On reaching their destination in this journey, the participants were looking forward to a fuller life beyond mere existence.

#### Theme 2: Building peer and therapeutic relationships

The findings strongly indicate the value of building peer and therapeutic relationships as part of the ePEEP journey (Table 5). The fellow travellers (peers and facilitator) on the journey enhanced learning and enabled change through shared experiences and validation. Participants valued that there was close personal virtual contact with the peers and the group facilitator, even though the program was offered remotely and online. The shared experience of pain contributed to the group cohesion. The participants valued that they could learn from each other; they felt supported and understood by each other and by the facilitators. There was a sense of belonging and accountability regarding goals on the journey of discovery. The peer-learning environment appears to have been successfully created in the virtual space with participants reporting that they felt safe engaging and communicating with the facilitator and with their peers. Moreover, the facilitators were successful in creating online therapeutic alliances.

**Table 5:** Theme 2: Building peer and therapeutic relationships

	Categories	Codes	Substantiating verbatim quote
THEME 2: Building peer and therapeutic relationships	Shared experience Group cohesion Collaborative peer relationships	Support Understanding Validation Accountability Responsibility	*We could really talk to each other openly and ask advice. We encouraged and strengthened each other. Our biggest role was to support each other. We did not know each other personally, but we understood each other. Gr2_P1* *We can relate to each other. It is nice to talk to someone that has the same problems that you are having. Because sometimes you speak to the doctor, they don’t really understand and some of them can be like oh, I’m exaggerating and stuff like that. Gr2_P5* *Because if we spoke about how we felt at home nobody would really understand because nobody has chronic pain but, in the group, we had everybody else experiencing chronic pain and he gave us the platform to share that with each other. So, I think it was that platform that was vital for me. Gr1_P4* *Because you want to participate in the group, you don’t want to be the odd one out and say I can’t do it. So, you have to at least try to do it. Gr2_P1*
	Relating in the online environment	Feeling connected Real-time interaction Therapeutic relationship	*I think it was better that way because I’m a very shy person when it comes to face-to-face and I think on the WhatsApp group it was much better because you can see that person, but you are not there exactly Gr2_P5* *The face time was what made it. And his preparation is what made it. It was always on key, on point, always ready. There was never a dull moment. We cried on the group, we laughed on the group, we shared on the group. Gr1_P3* *He (the facilitator) listens. He makes you feel like a person, he understands you, he has patience, he makes the time to help you understand if you don’t understand and he will agree to disagree with you if need be. Gr1_P3* *(The facilitators) played a huge role in our mindsets. We owe the thanks to them for the way they facilitated the program. Not only as a facilitator but also as somebody that understood what we were going through, somebody that was there to offer support and guidance. Gr1_P4*
	The need for personal contact	In-person session	*If it is in any way possible to do an actual session where people are together, so maybe if you are say for instance 5 in the group with your facilitator. You don’t have to physically touch but I feel that the experience would be different if you actually had that physical interaction with people. If it’s not an inconvenience for them to travel. The home thing was quite convenient but in my case, I would actually have preferred the physical interaction a bit more*
	The impact of non-completers	Abandonment Disruption Commitment Availability	*I felt like why do you make yourself available to participate and then not, because I’m the type of person and I just have a mindset where I promised I’m going to do this and I’m still doing it. Even though sometimes I do neglect doing some stuff, but you don’t start something and just leave it halfway because how do you know it’s going to help for you if you don’t finish it Gr2_P5* *And our first sessions, the first couple of attempts was a disaster because people were then available, then they were not available, when we started there wasn’t enough people, so it started touch and go. I felt, I literally they were going to cancel it because the people were not pitching and then eventually it started, Gr3_P6*

One of the main recommendations from the participants was that they would like some in-person time with their fellow travellers. Suggestions included an initial in-person meeting to facilitate forming the group, or a final meeting to reflect on the journey and successes. The value of such a meeting was proposed to strengthen relationships, learning and behaviour change. There were some participants (Fig. 1) who did not complete their ePEEP journey. The remaining participants noted that group cohesiveness was disturbed when a fellow traveller, with whom they started their journey, left the group prematurely. The latter indicates a disruption in group dynamics and relationship.

#### Theme 3: The online learning environment

Learning in the online environment was a novel experience for the participants. Learning in this way meant that travel on the journey of discovery was not straightforward, with some barriers forming bumps in the road, while numerous facilitators made the path easier and more enjoyable. Facilitators to learning comprised accessibility and convenience, safety, fun, provision of access, and the unique features of the online environment. The barriers to learning involved issues with internet connectivity, disturbances in the environment, user competence and that some who initially participated in the program, discontinued. Table 6 provides an overview of the factors that influenced learning in the online environment.

**Table 6:** Theme 3: Learning in the online environment

**Theme**	**Category**	**Code**	
**THEME 3: LEARNING IN THE ONLINE ENVIRONMENT**	Accessibility Convenience	Work integration Home responsibilities Travel	*It was good for me to receive it at home, even if I was at work, I would take time off and I would lock myself in an office and put my earphones and I would do it at work if I was not able to do it at home. It was convenient with the fact that I didn’t have to travel to all the things. Gr1_P4* *That was nice that it happened during lockdown because all my kids were home so it wasn’t a case of when I had my video calls that I would need to completely forget about them. They were all there, they could all support and be with each other while I had my sessions. Gr3_P6* *It was different in terms of where you individually have people that have to go to a hospital, people who have to travel, didn’t have to go out of their space to be part of it. So that to me was amazing so I didn’t find that at all challenging. Gr3_P6* *And that they gave you the data meant that you didn’t have to worry about am I going to have money to buy data, am I going to be able to participate in the group this week. So that goes hand in hand. It just made it, it is convenient. Gr3_P6*
	Safety	Emotional safety COVID safety	*I enjoyed the interaction the most. So, it was very comforting for me to see that I am not alone, there were other people experiencing pain as well, and there were other people that I could relate to. So, I think that was the best part, having people that I can relate to as well. Gr1_P4* *I felt very good because for me it is something that I needed and at the time I just felt with the risk of going to hospital I wasn’t prepared to go there and if I could do it on WhatsApp it will be much better for me. Gr2_P5*
	Fun	Laughter Joyful	*You know, it was such fun. Like we would exercise and someone would do something silly, we will not make a joke out of it but we all would laugh together just for the fun of it, but not like pick on the one at all – it was very joyful. For me it was like fun, joyful and you are healthy in doing, and just like doing it with other people it’s like, almost like it’s more encouragement for you to keep at this. Gr2_P5* *It was funny, but it was helpful. You know where you can let your hair down and just not worry for a couple of moments. That was the nice thing about the physiotherapist, and she would tell you how far to push yourself, how far not to push yourself. Gr3_P6*
	The technology	Data bundles Data sponsorship	*I think if you take anybody into consideration with, especially because it was lockdown and things were so horrible, that mere gesture of I will give you data so that you can be part of the group so that we can assist you to manage your pain better, it was just, I have no words to describe the positivity of it. Gr3_P6* *I had no money but they provided me with 2G data every Monday, so I had no problem with data. I had data to do everything they needed me to do and the video calls with group members individually if they needed my support, and to post my videos and I had no problem with my internet connection. Gr1_P3* *It was really nice because like if I had to do it on WhatsApp and they didn’t supply me with data I don’t think I would have been able to participate in it. Gr2_P5*
		User competence	*No. I think the older people in our group had difficulty in dealing or handling their video calls… I noticed that our physiotherapist and our psychologist, they struggled a lot to explain to the older people how to better their signal. Gr3_P6*
		Technology training	*I think when they do this with people and especially with older people, they need to …I think they should explain to them just send them a little video, just explain to them? Like all the exercises just show them how to mute the voice so that they can hear you, but we don’t necessarily hear them which means everything in the background doesn’t echo on the phone, or how to disconnect their the visual and still hear what’s happening. So, if there was a video they could create, especially the older ones, so that they know how to, it will make the process much easier. Gr3_P6*
	Impact of the home environment	Background noise Family circumstances	*It was very nice but sometimes it was a little bit of a challenge getting the kids not to make a noise, so some days – my mom stays around the corner from me – so when I know it is busy by me, I will go to my mom’s house just to get quietness sometimes, but it was very nice – I enjoyed it. Gr2_P5*
	The workbook	Writing Order of information	*Because I love the book, I love writing in my book, doing my homework, I just hated that if I was supposed to be on page 25 then I would actually end up on page 9 and 10 because that is where I thought that was the base of what you were learning, but then it was supposed to not be on that page. So it was, it was a bit confusing about what the right sections are. But what I loved about the material that we got. Gr3_P6*
	Course organisation	Duration of course	*The only thing that I would suggest is maybe for the program just to be a bit longer. We are currently doing the 6-week program. So maybe for each session have two weeks per module. So maybe 2 weeks per module. I think it would assist us in sticking to our goals and maintaining that balance where you can live life with the pain but also doing things that would lessen your pain. Gr1_P4*
		Follow up	*You know what I would suggest, I liked everything about it. Like even now the group is finished if they would still like, just now and then, just do a check-up and maybe have a group exercise again, because like you know, some of us may not continue b Gr2_P5*

Providing ePEEP facilitated greater access to care. Participants who were working, or who were responsible for childcare at home during lockdown, found the online environment more convenient compared to in-person sessions. The provision of data packages was a major facilitator to participation since it allowed participants to engage in the learning activities despite financial challenges and it eliminated travel costs. The provision of data packages contributed to adherence and allowed participants to continue travelling on their journey with consistency. Additionally, participating in ePEEP was perceived as safe, both from an emotional aspect and in terms of risk of exposure to COVID-19. The learning activities were experienced as fun and enjoyable, and participants particularly highlighted the fun of the exercise sessions. Participants further valued the online environment due to its flexible structure, the synchronous and asynchronous activities such as the front- and back-loading of podcasts and videos, and the ongoing input from the facilitator between live sessions.

Participants reported some challenges and distractions along the journey of discovery ranging from the challenge of learning online, family responsibilities, and the home environment. Distractions included background noise from their own and other participants’ home environments. The most frequently experienced challenge to participation in ePEEP was poor internet connectivity interfering with real-time interaction. While providing participants with data was helpful, poor cell phone coverage and signal quality was experienced by all. This meant that the facilitator and participants had to make alternative arrangements when video calls were not possible. Similarly, user competence, while a potential roadblock on the journey of discovery, was addressed with some assistance. Participants acknowledged that they or other participants might need more support to be able to optimise technology use and engage in the online learning environment. Congruently, study participants acknowledged that prior to starting, some participants may have benefited from better technology preparation and training to enable them to get the most out of the journey of discovery. Furthermore, the participants consistently recommended more course time to develop relationships, embed learning, and benefit from the relationships formed through their journey.

## Discussion

This study, to the knowledge of the authors, is the first reporting on the development, implementation, and evaluation of a group-based telehealth intervention for patients with chronic pain in the (South) African context. This group intervention was found to be feasible, acceptable, accessible, safe, and valuable for study participants. ePEEP facilitated a journey of self-discovery, empowerment, relationship building and transformation in the participants. The key strengths of ePEEP were the group cohesion, experiential and non-judgmental learning environment, and the accessibility of the online initiative. The findings of this study are important, since they address the knowledge gap in the user (participant) experience regarding group telehealth for chronic pain [13, 14].

The research allowed an in-depth understanding of patients' experience and perspectives regarding ePEEP, which could be instrumental for the design, implementation and decision- making regarding future telehealth interventions. A significant finding of this study is the participants’ journey of personal discovery and transformation. The journey of discovery was facilitated by their engagement not only with the ePEEP learning material, but through engagement in relationship with fellow participants and facilitators in an online platform. ePEEP provided the participants the opportunity to engage within a community of practice and to participate in active and patient-centred learning activities leading to them feeling empowered with new skills and knowledge. These findings concur with those of a group telehealth CPMP that was beneficial regarding pain-related outcomes and group cohesion in a rural Australian population [13]. Group interventions can be successfully delivered via telehealth with participants feeling actively engaged and supported [23]. Playing an active role in health care was found to be influential in reducing help seeking behaviour, the usage of healthcare services, and in improving quality of life. Additionally, Smith et al*.*, (2019) [24] found telehealth interventions for chronic pain superior to usual care for increasing pain self-efficacy. However, our study participants consisted only of women, and while pain rehabilitation programs have rendered positive outcomes for both men and women, gender differences should be acknowledged and further investigated [25]. Nonetheless, our study adds to the body of knowledge that confirms the efficacy of telehealth interventions, providing evidence for group based and interdisciplinary telehealth for chronic pain. The study findings contribute to the understanding of patients’ perspectives on *how* this change in outcomes occurs.

The valued features of ePEEP that facilitated change were the creation of therapeutic relationships, peer and facilitator support, and the learning environment. These relationships enhanced the journey of discovery. Correspondingly, most of the participants longed to have some in-person contact with group members and the facilitator. The importance of in-person contact to the participants indicates that a hybrid model of care may be preferable, and that not all chronic pain care can/should be delivered remotely [1, 13]. The group processes and real-time video interaction mitigated the effects of social isolation [13]. It is notable that the facilitators were able to create therapeutic alliances, trusting relationships, and a supportive learning environment, despite never having met the participants in person. The above finding contrasts with other studies that report patients struggling to connect with health care professionals in a telehealth environment when there wasn’t a pre-existing relationship [1, 2]. Congruently, it is recommended that group facilitators for telehealth initiatives be equipped with a core set of competencies which could include acquaintance with the technology, explaining chronic pain and teaching activity pacing [15], to optimize the learning environment and formation of the therapeutic relationship.

The intervention was patient-centred and considered participants' unique needs, preferences, and circumstances. The above was facilitated by the provision of individual and personal contact, via the asynchronous learning activities which could be completed at the participants preferred time and combined with synchronous real-time video interaction sessions. ePEEP enabled experiential learning through an information booklet with worksheets, goal setting exercises, and participation in relaxation and physical activity sessions. Although there was initial concern with initiating physical activity in the online setting, safe implementation was possible since a thorough evaluation of physical ability was done prior to referral. Congruently, exercise via telehealth has been implemented safely before for patients with chronic pain conditions [11, 13] and physical disabilities [26]. The guided problem-solving, patient-centredness and collaborative decision-making indicate key features for successful implementation of telehealth initiatives for chronic pain [1, 26].

Barriers to implementation were similar to those reported elsewhere on telehealth implementation [13, 15, 27], namely technology, internet access, and digital literacy; however, these barriers could be overcome. The collaborative problem-solving to address barriers in the group by adaptation of what was planned, formed a core part of the learning experience to guide problem-solving. This learning experience on adaptation and being flexible became part of the participants’ goal setting and narrative. The provision of data packages was a major facilitator to participation addressing financial inequities. It is therefore recommended this be a key consideration for future implementation of similar interventions in South Africa and other resource poor settings. The need for educational initiatives to optimise participants’ digital technology usage was also highlighted in the current study. Due to the nature of the study being exploratory, we excluded the three potential participants who did not have smartphones. To broaden access, and provide equitable opportunity to participate in telerehabilitation interventions, solutions should be sought for those who do not own smart phones, to bridge the digital divide. Congruently, Tauben et al*.*, (2020) [15] advises that subsidizing broadband and electronic equipment for telehealth initiatives may be required for success.

## Strengths and limitations

ePEEP allowed a group of strangers to collectively embark on a journey of discovery at a time when isolation and inertia were prominent features of daily life. They travelled on this journey with support and fun, challenging themselves along the way and successfully overcoming barriers to reach the destination of a transformed self. The telehealth initiative overcame barriers to care such as economic and transport concerns, geographic barriers, COVID-19 safety concerns, as well as access to care during the COVID-19 lockdown. ePEEP was therefore an effective mechanism to provide support to patients and to mitigate consequences of limited access for chronic pain [1]. There are indications that ePEEP offered unique and broadened learning opportunities when compared with in-person sessions, namely the opportunity for family involvement, access and use of resources that were distributed, peer led online support, and preparation for access to community-based groups.

Although ePEEP was initiated as part of adjusted measures to continue care for patients with chronic pain during the COVID-19 lockdown period, it has important implications for delivering rural and remote pain services. Those who are responsible for childcare or the care of relatives, may particularly benefit from the intervention due to its accessibility. In South Africa, people often travel long distances and/or over rough terrain to access pain care services which are largely only available in some tertiary metropolitan centres for a limited number of patients. Telehealth has the potential to increase quality and accessibility of health care, thereby contributing to equity in health care delivery in the African context [2, 28]. The study has provided several indications about the value of telehealth for chronic pain rehabilitation programs and indicates that telehealth has cemented itself as a viable option in the South African context. Telehealth should therefore be considered for inclusion in under- and postgraduate curricula. Additionally, health policies, clinical guidelines and care pathways should consider and include telehealth to enhance its uptake into practice [27].

More research is required regarding clinical outcomes of this telehealth initiative, since we only investigated fidelity, participant engagement, and user experience. Our research was a case study to investigate the feasibility of ePEEP, therefore, the results can only be generalised to similar contexts. The study sample size was small, and based on self-selection, which further impacts generalisability; however, data saturation was achieved. One of the major considerations is the gender bias in our study. The study focusses on the perspectives of women who participated in the group initiative. Based on the information that only one male, out of the total population eligible to participate were interested in participating, more research exploring the role of gender on participation in group interventions would be valuable [25]. We highlighted several lessons to be learnt from our implementation and investigation. It is suggested that more information be gained from patients who did not start or did not complete the program to ascertain reasons for non-participation. Such information would assist to optimize the ePEEP initiative. Although we attempted to contact these individuals, they could not be reached telephonically or declined to participate. ePEEP requires access to a mobile smart device with a camera, microphone and speaker technology and mobile data. These may not be accessible to all patients and may have to be provided or subsidized. The main suggestions to optimize the ePEEP of the future was to consider the duration of the interventions, to include at least one in-person session, and to address digital literacy prior the start of ePEEP.

## Conclusion

The telehealth group intervention that was developed and implemented, was found to be acceptable, accessible, safe, and valuable for the participants. ePEEP facilitated self-discovery, empowerment, and transformation in the participants, through experiential learning, therapeutic relationships, social and facilitator support, and peer-assisted learning. The subsidized mobile data allowance was a major facilitator to participation. Future telehealth innovations in this context should include the monitoring of clinical outcomes and an in-person group session, when feasible.

## Supplementary Information

**Additional file 1.**

## Data Availability

The datasets used and/or analysed during the current study are available from the corresponding author on reasonable request. The datasets generated and/or analysed during the current study are not publicly available due to their nature as qualitative interview data but are available from the corresponding author on reasonable request.

## References

[1] Eccleston C, Blyth FM, Dear BF, Fisher EA, Keefe FJ, Lynch ME, Palermo TM, Reid MC, Williams ACC (2020). Managing patients with chronic pain during the COVID-19 outbreak: considerations for the rapid introduction of remotely supported (eHealth) pain management services. Pain.

[2] Dassieu L, Pagé MG, Lacasse A, Laflamme M, Perron V, Janelle-Montcalm A, Hudspith M, Moor G, Sutton K, Thompson JM (2021). Chronic pain experience and health inequities during the COVID-19 pandemic in Canada: qualitative findings from the chronic pain & COVID-19 pan-Canadian study. Int J Equity Health.

[3] Fallon N, Brown C, Twiddy H, Brian E, Frank B, Nurmikko T, Stancak A (2021). Adverse effects of COVID-19-related lockdown on pain, physical activity and psychological well-being in people with chronic pain. Br J Pain.

[4] Javed S, Hung J, Huh BK (2020). Impact of COVID-19 on chronic pain patients: a pain physician's perspective. Pain Manag.

[5] Dueñas M, Ojeda B, Salazar A, Mico JA, Failde I (2016). A review of chronic pain impact on patients, their social environment and the health care system. J Pain Res.

[6] Scascighini L, Toma V, Dober-Spielmann S, Sprott H (2008). Multidisciplinary treatment for chronic pain: a systematic review of interventions and outcomes. Rheumatology (Oxford).

[7] Kamerman PR, Bradshaw D, Laubscher R, Pillay-van Wyk V, Gray GE, Mitchell D, Chetty S (2020). Almost 1 in 5 South African adults have chronic pain: a prevalence study conducted in a large nationally representative sample. Pain.

[8] Parker R, Burgess S, Dubaniewicz A, Gouws L, Krone J, Madden V, Nortje C, Parsons C (2009). Patient satisfaction with a pilot chronic pain management programme in Cape Town. South Africa. S Afr J Physiother.

[9] Sharpe L, Jones E, Ashton-James CE, Nicholas MK, Refshauge K (2020). Necessary components of psychological treatment in pain management programs: A Delphi study. Eur J Pain.

[10] Wilson IR (2017). Management of chronic pain through pain management programmes. Br Med Bull.

[11] Adamse C, Dekker-Van Weering MG, van Etten-Jamaludin FS, Stuiver MM (2018). The effectiveness of exercise-based telemedicine on pain, physical activity and quality of life in the treatment of chronic pain: A systematic review. J Telemed Telecare.

[12] Buhrman M, Gordh T, Andersson G (2016). Internet interventions for chronic pain including headache: A systematic review. Internet Interv.

[13] Scriven H, Doherty DP, Ward EC (2019). Evaluation of a multisite telehealth group model for persistent pain management for rural/remote participants. Rural Remote Health.

[14] Fernandes LG, Devan H, Kamper SJ, Williams CM, Saragiotto BT (2020). Enablers and barriers of people with chronic musculoskeletal pain for engaging in telehealth interventions: protocol for a qualitative systematic review and meta-synthesis. Syst Rev.

[15] Tauben DJ, Langford DJ, Sturgeon JA, Rundell SD, Towle C, Bockman C, Nicholas M (2020). Optimizing telehealth pain care after COVID-19. Pain.

[16] Korstjens I, Moser A (2017). Series: Practical guidance to qualitative research. Part 2: Context, research questions and designs. Eur J Gen Pract.

[17] Moser A, Korstjens I (2018). Series: Practical guidance to qualitative research. Part 3: Sampling, data collection and analysis. Eur J Gen Pract.

[18] Kruse CS, Krowski N, Rodriguez B, Tran L, Vela J, Brooks M (2017). Telehealth and patient satisfaction: a systematic review and narrative analysis. BMJ Open.

[19] LeRouge CM, Garfield MJ, Hevner AR (2015). Patient perspectives of telemedicine quality. Patient Prefer Adherence.

[20] Stats SA General Household Survey 2018 Pretoria Statistical Release PO318. Available at: https://www.statssa.gov.za/publications/P0318/P03182019.pdf.

[21] Braun V, Clarke V (2006). Using thematic analysis in psychology. Qual Res Psychol.

[22] Frambach JM, van der Vleuten CP, Durning SJ (2013). AM last page. Quality criteria in qualitative and quantitative research. Acad Med.

[23] Barello S, Triberti S, Graffigna G, Libreri C, Serino S, Hibbard J, Riva G (2016). eHealth for Patient Engagement: A Systematic Review. Front Psychol.

[24] Smith J, Faux SG, Gardner T, Hobbs MJ, James MA, Joubert AE, Kladnitski N, Newby JM, Schultz R, Shiner CT (2019). Reboot Online: A Randomized Controlled Trial Comparing an Online Multidisciplinary Pain Management Program with Usual Care for Chronic Pain. Pain Med.

[25] Pester BD, Crouch TB, Christon L, Rodes J, Wedin S, Kilpatrick R, Pester MS, Borckardt J, Barth K (2022). Gender differences in multidisciplinary pain rehabilitation: The mediating role of pain acceptance. J Contextual Behav Sci.

[26] Devan H, Hale L, Hempel D, Saipe B, Perry MA (2018). What Works and Does Not Work in a Self-Management Intervention for People With Chronic Pain? Qualitative Systematic Review and Meta-Synthesis. Phys Ther.

[27] Birnie KA, Killackey T, Stinson J, Noel M, Lorenzetti DL, Marianayagam J, Jordan I, Jordan E, Neville A, Pavlova M (2021). Best practices for virtual care to support youth with chronic pain and their families: a rapid systematic review to inform health care and policy during COVID-19 and beyond. Pain Rep.

[28] Chifamba N (2018). A scoping review on the challenges of Telemedicine implementation the Southern Africa.

