# Supplementary material for: “So, you must understand that that group changed everything”: perspectives on a telehealth group intervention for individuals with chronic pain
Source: BMC Musculoskelet Disord. 2022 Jun 4;23:538. doi: 10.1186/s12891-022-05467-7 (PMC9166594; doi:10.1186/s12891-022-05467-7)
Supplement: Supplementary file 1 — Additional file 1. [file 12891_2022_5467_MOESM1_ESM.docx]

**Introduction: aim, informed consent, rights of the participant**

**Thank you for being willing to participate in the interview.**

**Opening question:**

- - - 1. Offering the ePEEP was a new experience, since this was the first time that it was offered through TBH and GSH. What was it like for you to be part of this new venture?

**Core questions:**

- - - 1. Could you tell me about your expectations for that very 1^st^ ePEEP contact?
      2. Did your expectations change during the course of ePEEP?

*If so, please elaborate*

- - - 1. In your view, what was the most challenging part of facilitating/presenting PEEP in the telehealth format?
      2. Could you suggest /elaborate on potential solutions for the challenges you mentioned?
      3. Which parts of ePEEP worked really well in your group? Please elaborate.
      4. Please share with me your thoughts regarding the patients’ role in ePEEP?
      5. Do you have any suggestions how e-PEEP can be optimised for the SA context?
      6. If you had to provide advice for clinicians embarking on offering telehealth for chronic pain management, what would your key messages be?

*Probe for contextual factors (person, environment, system and technology)*

**Closing question**

- - - 1. Do you have any comments or questions that you think would be useful for the ePEEP team to consider?

**Thank you for your contribution!**

**Next steps and how participants will be informed of the outcome.**
